# Transcatheter Aortic Valve Implantation Followed by Lung Cancer Resection in an Older Patient: A Case Report

**DOI:** 10.7759/cureus.118353

**Published:** 2026-10-22

**Authors:** Leena Khan, Syed S Qadri

**Affiliations:** 1 General Practice, West Rainton Surgery, County Durham and Darlington NHS Foundation Trust, Durham, GBR; 2 Thoracic Surgery, Hull University Teaching Hospitals NHS Trust, Hull, GBR

**Keywords:** aortic stenosis, lobectomy, lung cancer, transcatheter aortic valve implantation, video-assisted thoracoscopic surgery

## Abstract

The individual incidences of lung cancer and cardiovascular disease in the elderly population mean that there is a strong likelihood of both occurring simultaneously. Common practice for severe aortic stenosis (AS) would be surgical aortic valve replacement (AVR), unless deemed high-risk for this surgical procedure. Age and lung cancer both increase a patient’s surgical risk; hence, the less invasive transcatheter aortic valve implantation (TAVI) to manage valve disease ahead of lung resection for lung cancer is an acceptable alternative after assessing other factors such as procedural risk, comorbidities, and prognosis. We present the case of an 82-year-old female patient who underwent a TAVI seven months before lung resection for an incidental diagnosis of lung cancer with staging T1b N0 M0. Postoperatively, she was treated for atrial fibrillation and lung collapse, then discharged after resolution of these complications on the eighth day after surgery, and remained well at follow-up three months later. This case report explores a situation when this sequence of interventions was appropriate due to increased surgical risk in the elderly population.

## Introduction

Lung cancer is reported to be a primary cause of cancer-related deaths globally, according to the World Health Organization (WHO) [1]. With the incidence of lung cancer increasing with age due to the risk of cumulative smoking burden, patients are presenting with multiple comorbidities, including aortic stenosis (AS) [2]. 

Typically, surgical aortic valve replacement (AVR) is the choice of intervention for severe symptomatic AS prior to non-cardiac surgery according to the European Society of Cardiology (ESC) and European Association for Cardio-Thoracic Surgery (EACTS) after considering surgical risk and clinical urgency [3]. In high-risk and comorbid patients who are not fit for AVR, a less invasive transcatheter aortic valve implantation (TAVI) is recommended before non-cardiac surgery [3]. 

There are limited cases published using this two-stage approach; thus, this case report follows a high-risk patient with severe AS and lung cancer who underwent a TAVI prior to a lobectomy to manage both diagnoses in the lowest-risk manner. 

## Case presentation

The patient was an 82-year-old female who presented to our hospital with a PET-positive lesion in the right upper lobe of the lung. This lesion was an incidental finding at the time of a TAVI workup (Figure 1). 

**Figure 1:**
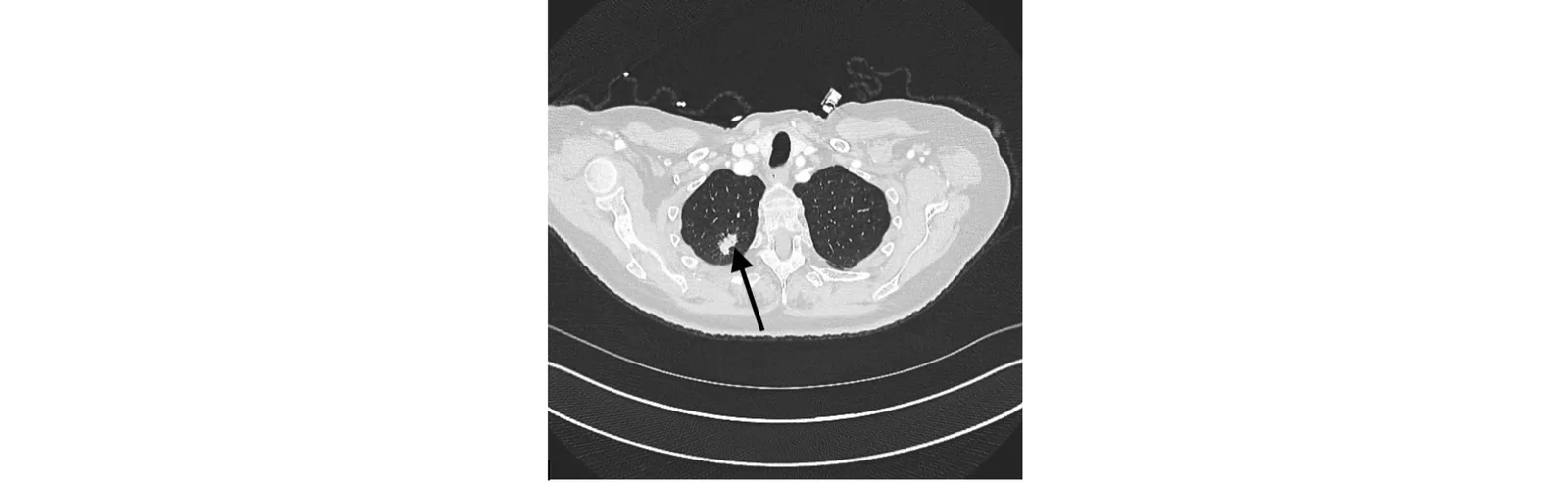
CT chest showing right upper lobe lung tumor (arrow).

In February 2025, she was found to have severe aortic valve calcification on an echocardiogram with normal left ventricular function. Although she was asymptomatic at the time, she underwent a TAVI in April 2025, performed in a hospital across the country, as she was deemed unfit for an AVR due to age and comorbidities, as per documentation available to the authors; however, the documentation has not revealed the indication for intervention at this stage. Her relevant past medical history included emphysema, coronary artery disease, and a 75 pack-year smoking history. The TAVI occurred without complication as reported in the hospital notes, but the patient became generally deconditioned as a result of her hospital stay.

After recovering from the TAVI, she underwent investigation of the incidental lung lesion, which was a PET-positive right upper lobe lung lesion. Subsequently, a CT-guided biopsy confirmed a squamous cell carcinoma with staging T1b N0 M0. She remained asymptomatic from this tumor with an Eastern Cooperative Oncology Group (ECOG) performance status of 1 [4]. She was listed for a right upper lobectomy. 

Seven months after her valve procedure, to allow time for adequate recovery after TAVI, to regain baseline functional status, and for preoperative assessments, she underwent a bronchoscopy and right video-assisted thoracoscopic surgery (VATS) and right upper lobectomy and lymph node dissection in November 2025. The surgery occurred without intraoperative complications. The bronchus to the right upper lobe was transected after surrounding vessels and structures were dissected. The right upper lobectomy was performed, then hemostasis was achieved, and a 28Fr chest drain was inserted to the apex as the lung was reinflated. She returned to the surgical ward with a Thopaz chest drain in situ.

Her chest drain was removed on day one postoperatively, and a subsequent chest X-ray showed a small right pleural effusion, which persisted, as did her oxygen requirement of four liters despite chest physiotherapy. A CT chest showed right middle lobe collapse and partial right lower lobe collapse with mucous plugging. She later developed new atrial fibrillation at a heart rate of 150 bpm. An arterial blood gas (ABG) on four liters of oxygen showed type 1 respiratory failure with a pO2 of 10.5 kPa and a pCO2 of 5.2 kPa. She was treated with 40 mg verapamil and transferred to the ICU for type 1 respiratory failure due to right lung collapse requiring high-flow nasal oxygen. After stabilization and weaning off high-flow nasal oxygen, she was stepped down to the ward, remaining on antibiotics for a lower respiratory tract infection and treated with regular 2.5 mg salbutamol nebulizers, 750 mg carbocisteine three times per day, and chest physiotherapy. For the remainder of her hospital stay, she was in normal sinus rhythm, and after repeat ABGs, her target oxygen saturations were 88-92%, given her background of emphysema. She engaged with physiotherapy to aid her recovery and was discharged on day eight after her surgery, with satisfactory resolution of lung changes on subsequent chest X-rays.

She had surgical follow-up in the Thoracic Clinic in March 2026. She had made a good recovery following surgery without recurrence of the tumor. 

## Discussion

AS is a form of valvular heart disease causing left ventricular (LV) obstruction and increased LV systolic pressure, common among the elderly population with a prevalence of 12.4% in those 75 years or older [5,6]. Since lung cancer incidence increases with age [2], the likelihood of having concurrent lung cancer and AS is much greater in the elderly population. It has been reported that 18% of lung nodules are found incidentally on CT scans during preoperative investigations for AS intervention [7]. 

The etiology of AS varies from congenital bicuspid valve to acquired causes including rheumatic disease, calcification, systemic lupus erythematosus, and metabolic disease. Despite the range of causes for acquired disease, the common presentation of AS includes exertional dyspnea, angina, syncope, and features of heart failure [5]. The severity of disease often does not correspond to the severity of symptoms, as severe AS can remain asymptomatic, as in the case of this patient.

The conventional management for symptomatic and severe asymptomatic AS is AVR, which would entail open-heart surgery with cardiopulmonary bypass. Many elderly patients would be deemed high-risk for this type of procedure, and concurrent lung cancer is associated with the risk of tumor dissemination [8,9]. Therefore, the less invasive method of TAVI would be preferable for such patients prior to lung resection of lung cancer, as it does not involve extracorporeal circulation. 

The choice between a two-surgery or concomitant surgical approach for lung and valve disease remains up for debate, as concomitant surgery has been shown to have a higher risk of postoperative complications compared to a two-surgery approach [10]. However, it can be argued that two separate surgeries, with the valve disease being treated first, lead to delay and potential progression of lung cancer. Hence, the time interval between the two surgeries should consider adequate recovery time from TAVI, and the stage of malignancy and likelihood of progression of lung cancer. In our case, the patient had her pulmonary resection seven months after the TAVI. 

Other cases have undergone thoracic surgery after shorter intervals since TAVI. Drevet et al. present the case of a 75-year-old man who underwent a TAVI for severe AS, then a lobectomy for left upper lobe squamous cell carcinoma approximately 72 hours later. Postoperatively, he suffered from atrial fibrillation and pneumonia, like this patient, but has not had a recurrence of lung cancer after one year [11]. 

As can be seen from this, the risk of complications between the case above and this patient is similar; yet, this patient had adequate time to recover after the initial valve intervention. However, further cases have reported that shorter intervals between the two procedures can be optimal given that TAVI is much less invasive than the traditional AVR surgery and allows for faster recovery post-procedure [12]. 

Although this case report presents valuable information for this two-stage approach, the limitations of the report largely include the lack of some key details in the documentation available to the authors at the time of writing, including the complete echocardiogram details to explain the severity of AS aside from the level of calcification, and the absence of EuroSCORE II [13] or anatomical details prior to TAVI to objectively support the patient’s surgical risk and indication for the TAVI. Furthermore, the full histopathology results were not available.

## Conclusions

As the incidence of concurrent AS and lung cancer will likely increase, we suggest that a two-stage surgical approach with TAVI followed by a lobectomy is a sensible and lower-risk strategy to manage such patients, as it avoids the need for the more invasive cardiopulmonary bypass and reduces the risk of tumor dissemination. The elderly cohort that this report focuses on tends to be higher-risk candidates for surgery; therefore, we should adopt the lowest-risk approach for management. However, only a few cases of this strategy are documented in the literature, and the optimal time between the two procedures is still debated.
